# Enhancing resolution and image quality in musculoskeletal MRI using deep learning reconstruction

**DOI:** 10.1186/s41747-026-00743-w

**Published:** 2026-05-28

**Authors:** Marco Porta, Giuseppe Agresti, Maria Marcella Laganà, Stefano Orofino, Sara Pangaro, Nicola Carapella, Pietro Andrea Bonaffini, Paolo Bernasconi, Eugenio Annibale Genovese, Sandro Sironi, Alberto Aliprandi

**Affiliations:** 1 Department of Radiology, Istituti Clinici Zucchi, Monza (MB), Italy; 2 Canon Medical Systems, Rome, Italy; 3 Department of Radiology, University of Brescia, Brescia, Italy; 4 Department of Radiology, ASST Papa Giovanni XXIII Hospital, Bergamo, Italy; 5 School of Medicine, University Milano Bicocca, Milano, Italy; 6 Studio Radiologico Bernasconi, Seregno (MB), Italy; 7 Medicine and Surgery Department, Insubria University, Varese, Italy; 8 Medical Clinical Institute Intermedica–Columbus, Milano, Italy

**Keywords:** Deep learning, Image processing (computer-assisted), Magnetic resonance imaging, Musculoskeletal system, Signal-to-noise ratio

## Abstract

**Objective:**

Deep learning-based noise reduction enhances image quality, overcoming the tradeoff among acquisition time, spatial resolution, and signal-to-noise ratio (SNR). We implemented deep learning reconstruction (DLR) into a 1.5-T musculoskeletal (MSK) magnetic resonance imaging (MRI) protocol to improve image quality without compromising SNR.

**Materials and methods:**

We retrospectively analyzed 39 MRI examinations performed on a 1.5-T scanner using standard-resolution (SR) sequences and sequences with higher resolution reconstructed with DLR (HR-DLR). Exams of the knees, shoulders, ankles, and hips were evaluated. The included sequences were: three-dimensional T2-weighted fast advanced spin-echo; T1-weighted and proton density-weighted fast spin-echo. One expert reader and two junior readers in agreement evaluated the visibility of various structures using a 5-point Likert scale in a blind manner. A fourth reader estimated the SNR and contrast-to-noise ratio (CNR) in bone and muscle. A mixed model was used to compare HR-DLR *versus* SR measures. The agreement between radiologists was assessed with the Kendall τ coefficient.

**Results:**

The HR-DLR sequences globally had a smaller pixel size and shorter acquisition time. A good inter-reader agreement was obtained for SR sequences (0.613 ≤ τ ≤ 0.788) and even higher levels of agreement for HR-DLR sequences (0.682 ≤ τ ≤ 0.961). All the structures had higher or similar Likert scores in HR-DLR sequences (*p* < 0.001), regardless of joint and sequence contrast. Apparent SNR and CNR of HR-DLR and SR were similar.

**Conclusion:**

Incorporating DLR into 1.5-T MSK MRI protocols enhances resolution and maintains SNR and CNR, improving MSK structure visualization.

**Relevance statement:**

This study demonstrated the effectiveness of deep learning reconstruction in improving the efficiency of 1.5-T musculoskeletal MRI exams. Despite shorter acquisition times, the visibility of key MSK structures was consistently rated as superior or similar in higher-resolution images with DLR.

**Key Points:**

- MRI is one of the primary diagnostic tools for evaluating MSK injuries and disorders.
- Deep learning reconstruction (DLR) implemented in a 1.5-T MSK protocol improved resolution, still preserving SNR and CNR.
- Higher scores were assigned by different raters to the DLR images, showing image quality improvement.

**Graphical Abstract:**

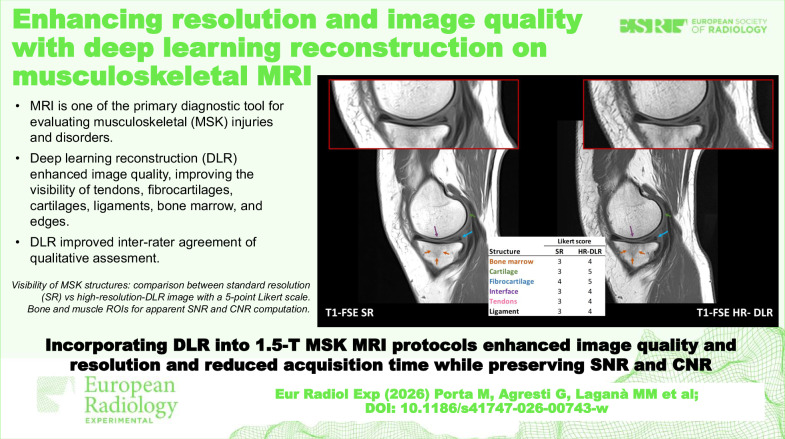

## Background

Magnetic resonance imaging (MRI) is one of the primary diagnostic tools for evaluating musculoskeletal (MSK) injuries, offering high-resolution images and exceptional soft tissue contrast [1]. However, long acquisition times limit patient throughput, increase discomfort, raise the likelihood of motion artifacts, and elevate costs [2–5]. These limitations have driven the development of faster imaging techniques, aimed at improving the efficiency and accessibility of MRI for MSK diagnostics [4, 6, 7]. When reducing scan time, a tradeoff must be reached in terms of spatial resolution and signal-to-noise ratio (SNR). High-resolution (HR) images are crucial for fine anatomical details, but smaller voxel sizes inherently reduce the SNR. The latter can be restored with a higher number of excitations, yet prolonging acquisition time, possibly reducing patient comfort, and increasing the likelihood of motion artifacts and the need for rescans.

In recent years, artificial intelligence, in particular, deep learning-based reconstruction (DLR) methods, have emerged [2, 8] as effective tools for reducing noise, enabling shorter acquisitions [8, 9], enhancing image quality and increasing spatial resolution [10]. Numerous DLR solutions are already embedded in clinical MRI scanners [11], enhancing image quality, patient positioning and comfort, and workflow efficiency [12]. A recent statement on technology readiness levels for artificial intelligence tools in cardiac computed tomography and MRI classified the maturity of image acquisition and reconstruction as generally high [13]. Having DLR implemented as an on-scanner reconstruction tool allows its direct use in routine clinical practice, without additional processing time or workflow delays.

With DLR, several MSK studies have focused on scan time reduction. In particular, accelerated sequences [14–18], or acquisitions performed with a reduced number of signal averages [19, 20], when reconstructed using deep learning, demonstrated improved image quality and diagnostic performance compared to that of conventional protocols, despite the shorter acquisition time.

DLR has also been applied to increase resolution and reduce scan time, with preserved image quality [10, 21–23]. Do et al [10] showed that 3-T HR MSK sequences reconstructed with DLR achieved superior radiological quality compared to conventional methods, despite a decrease in SNR and CNR from halving voxel size. Similarly, Akai et al demonstrated that DLR improved image quality and structural visualization on 1.5-T proton density (PD)-weighted knee images, even with fewer averages [19, 20, 24]. While these studies focused exclusively on the knee, in this work, we extended the evaluation to multiple joints at 1.5 T, the most widely used field strength in European clinical practice.

Based on previous literature [10, 20, 24, 25], we first hypothesized that HR sequences, combined with DLR, would facilitate assessment of various MSK structures. Second, we hypothesized that improving the resolution to a lesser extent compared with Do et al [10], the apparent SNR in bone and muscle, as well as the CNR between bone and muscle, would be at least non-inferior in HR-DLR images compared to standard-resolution (SR) images.

Accordingly, this study aimed to evaluate whether: (1) DLR improves visualization of key MSK structures in higher-resolution, faster sequences; (2) the SNR and CNR are preserved in the HR-DLR sequences, despite the increased resolution and reduced acquisition time.

## Methods

This retrospective study was approved by the Ethical Committee. Since this study was a retrospective analysis based on irreversibly anonymized MRI, written informed consent from patients was waived.

### Subjects and MRI protocol

The study participants underwent clinical MSK MRI with a 1.5-T scanner (Vantage Elan, NX Edition; Canon Medical Systems) at a private clinic (Studio Radiologico Bernasconi S.r.L.).

The study included 42 subjects who were examined with paired sequences during the same exam session: a routine standard-resolution (SR) sequence and a higher-resolution sequence using “Advanced intelligent Clear IQ Engine”—AiCE (Canon Medical Systems) (HR-DLR sequence), both with the same type of contrast (T1, T2, or PD-weighting).

The DLR method used in the current study and by other research groups [19, 20, 24] is a convolutional neural network previously described by Kidoh et al [26]. This system operates directly on the MRI console, processing the acquired raw data immediately after acquisition for image reconstruction. By integrating image noise reduction into the reconstruction workflow, AiCE enhances image quality without requiring additional steps or external processing. The configuration of the DLR method for each sequence, along with the corresponding conventional acquisition parameters, is reported in Supplementary Table S1.

The retrospective dataset included examinations acquired during the initial implementation of DLR in clinical practice. During this phase, conventional and AiCE sequences with modified parameters (*e.g*., higher resolution and possibly shorter acquisition time) were acquired in parallel to allow radiologists to familiarize themselves with the new reconstruction method. This practice, adding only 2–3 min per scan, is commonly adopted for training purposes. DLR sequences were added according to scheduling feasibility, without preselection based on patient characteristics or expected compliance. The images were examined, and the following inclusion and exclusion criteria were applied.

We included subjects older than 18 years who underwent MRI of the knees, shoulders, ankles, and hips. Included contrast pairs were: T1-weighted fast spin-echo (T1-FSE); PD-weighted FSE with fat saturation (PD-FS); and 3D T2-weighted fast advanced spin-echo (FASE 3D-T2). Contrast pairs were excluded if even just one of the two acquisitions showed severe artifacts due to motion or prosthesis, or suboptimal fat saturation.

Technical parameters for the routine SR sequences and the HR-DLR ones are reported in Supplementary Table S1.

The following dedicated coils were used to examine the various anatomies: a 8-channel array knee coil (8ch Knee SPEEDER, Canon Medical Systems), a 6-channel array winged designed coil for the shoulder (Shoulder SPEEDER, Canon Medical Systems), a 16-channel flex coil (16ch Flex SPEEDER coil Medium, Canon Medical Systems) for the ankle, and a 16-element phased array body coil plus a 12-element phased array spine coil (Atlas SPEEDER Body and Octave SPEEDER Spine, Canon Medical Systems) for the hips.

### Qualitative evaluation

All the images were visualized on a Sectra Picture Archiving and Communication System (Linköping, Sweden). The visibility of various structures (tendons, fibrocartilages, cartilages, ligaments, bone marrow, and interfaces) was assessed for each joint. Fibrocartilages were not evaluated in the ankle, as this joint does not contain structures analogous to the menisci or labrum of the knee, hip, or shoulder.

For each structure, a 5-point Likert scale was used to globally evaluate the structure visibility, assessing the diagnostic quality [27]. The scale values were as follows: 1 = not visible/poor visibility; 2 = only partially visible; 3 = fully visible with blurring border; 4 = slight blurring borders; and 5 = excellent delineation.

All the subjects were evaluated by three readers: one with 20 years of radiological experience (R1, A.A.), and two with 4 years of radiological experience (R2,3) who gave a unique evaluation in consensus. The reading sessions performed by R1 and R2,3 were completely independent. All the scores were assigned blindly to the image information.

### Quantitative analysis

**Fig. 1:**
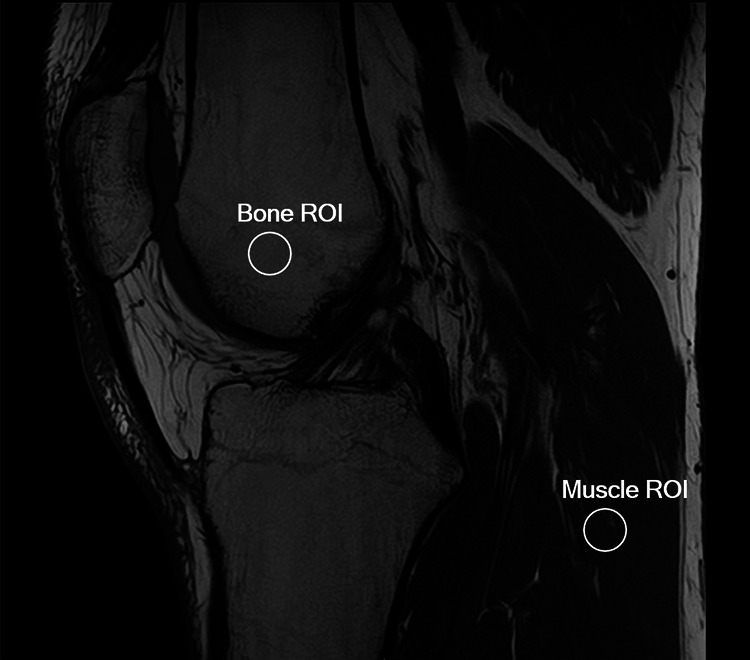
ROI positioning for the signal-to-noise ratio and contrast-to-noise ratio estimates: 1-cm diameter ROIs were manually positioned in the bone marrow and in the muscle

A fourth reader (G.A., radiology resident, 4 years of experience), blinded to the qualitative scores given by his colleagues, manually drew two 1-cm diameter circular regions of interest (ROI), as shown in Fig. 1: one in the bone marrow and another one in the muscle, for estimating their apparent SNR and apparent CNR.

Following the approach of Do et al [10], and considering that only a single acquisition was available for each dataset, as well as the known spatial heterogeneity of noise in parallel imaging and DLR reconstructions, SNR values were computed using a single-ROI method [28, 29]. We therefore reported this metric as apparent SNR, defined as the mean signal intensity within the ROI divided by its standard deviation.

The apparent CNR between bone marrow and muscle was estimated by normalizing the absolute difference between the average signals in the two ROIs to the standard deviation of signal intensities within the muscle ROI [10].

### Statistical analysis

All the statistical analyses were performed using Jamovi module version 2.3.28 (https://gamlj.github.io/).

The agreement between radiologists (R1 and R2,3) for the Likert scores of the various MSK structures was assessed with Kendall τ, separately for DLR and non-DLR sequences. with τ ranging from 0 (no agreement) to 1 (complete agreement). Paired scores were used for all the subjects, except for those who performed the ankle exam, where the fibrocartilage was not evaluated. The following classification was used for scoring the resulting agreements: < 0.200 = poor; 0.200‒0.400 = fair; 0.401–0.600 moderate; 0.601‒0.800 = good; and 0.801‒1.000 = very good [30].

Total acquisition time, and average pixel and voxel sizes (acquired and reconstructed ones) were computed considering all the sequences (T1-FSE, PD-FS, and FASE 3D-T2), separately for each joint. For each parameter, the percentage of change between the two protocols was also computed as:

$$\Delta \% = \frac{\text{SR \; sequence} - \mathrm{HR}{\mbox{-}}\text{DL \; R sequence}}{\text{SR \; sequence}}$$

In order to test if the higher-resolution sequences with DLR had different SNR, CNR and diagnostic Likert scores compared to the routine sequences, we evaluated the repeated (coupled) measures using linear mixed models (LMM). For each LMM, the independent variable was one of the qualitative or quantitative variables. The fixed variables were: DLR presence; joint (knees, shoulders, ankles and hips); sequence (PD-FS, T1-FSE, FASE 3D-T2); and the interactions between DLR presence and joint or sequence. A random effect was also included for each patient, allowing individual variability to be considered in the analysis.

After fitting the LMM, for the statistically significant interactions, the *post hoc* tests were assessed to further examine the interaction effects between joints and DLR presence, as well as between the sequence contrast and DLR. This approach helped to clarify how the effect of HR combined with DLR might change depending on the particular anatomical location (joint) or sequence contrast, allowing for a more detailed understanding of these interactions in the model. All the tests were corrected for multiple comparisons using Bonferroni’s correction. The *p*-values < 0.05 were considered statistically significant.

## Results

### Subjects

Forty-two patients were evaluated. One subject was excluded for movement artifacts in the SR acquisition, and two for sequences with contrasts different from those of the inclusion criteria. Thus, 39 subjects, 22 females and 17 males, with a median age of 52 years, range 18‒82 years, entered the analyses. Globally, 14 knees, 8 shoulders, 8 ankles, and 9 hips were examined, with 16 T1-FSE, 16 PD-FS, and 7 FASE 3D-T2 sequences.

### Protocol parameters

Table 1 shows the total acquisition time, average pixel and voxel sizes for the routine SR and the HR-DLR protocols, as well as the percentage change of the HR-DLR sequences compared to the SR ones. The results were grouped by joint.

**Table 1:** Total AT, average pixel and voxel sizes for the SR *versus* HR-DLR

		SR	HR-DLR	Δ% (SR *minus* HR-DLR)/SR
AT (sum across sequences) (ms)	Knee	586	597	-1.9%
	Shoulder	524	504	3.8%
	Hip	447	348	22.1%
	Ankle	365	278	23.8%
Average pixel size across sequences (mm^2^)	Knee	0.570	0.499	12.4%
	Shoulder	0.681	0.495	27.3%
	Hip	1.481	1.029	30.5%
	Ankle	0.294	0.212	27.9%
Average voxel size across sequences (mm^3^)	Knee	1.396	1.227	12.1%
	Shoulder	1.729	1.027	40.6%
	Hip	7.406	5.144	30.5%
	Ankle	0.882	0.636	27.9%
Average reconstructed pixel size across sequences (mm^2^)	Knee	0.144	0.126	12.8%
	Shoulder	0.172	0.124	27.9%
	Hip	0.374	0.259	31.0%
	Ankle	0.074	0.053	28.6%
Average reconstructed voxel size across sequences (mm^3^)	Knee	0.352	0.310	12.1%
	Shoulder	0.436	0.257	41.1%
	Hip	1.872	1.293	31.0%
	Ankle	0.223	0.159	28.6%

*AT* Acquisition time, *DLR* Deep learning reconstruction, *HR* High-resolution, *SR* Standard-resolution routine sequence as a reference

As expected for the adopted protocols, the pixel and voxel sizes of the HR-DLR sequences compared to the SR ones were always smaller. The reduction in pixel size ranged between 12% for the knee and 31% for the hip, with intermediate reductions observed for the other joints, as reported in Table 1. Similarly, the reduction in voxel size was between 12% for the knee and 41% for the shoulder, as reported in Table 1.

In addition, we had a reduced acquisition time, with values ranging from 3.8% (shoulder) to 23.8% (ankle) lower in HR-DLR compared to standard sequences and a slight increase of 1.9% for the knee (Table 1).

### Inter-rater agreement

Thirty-nine paired Likert scores (R1 *versus* R2,3) were used to compute Kendall’s τ coefficient for tendons, cartilages, ligaments, bone marrow, and interfaces. For fibrocartilage, 31 paired scores were used, since fibrocartilage was not evaluated in the ankle examinations (8 subjects). The radiologists’ agreements are reported in Table 2, with good agreement (τ range 0.613‒0.788) for SR sequences, and even better for DLR sequences, with good to very good agreement (τ 0.682‒0.961). For all correlations, *p*-values were lower than 0.001.

**Table 2:** Kendall τ of the correlation between the two scores, given independently by R1 and R2,3 for SR sequences and HR-DLR sequences

Structures	Number of paired Likert scores	SR Kendall τ	HR-DLR Kendall τ
Tendon	39	0.613	0.827
Fibrocartilage	31	0.730	0.800
Cartilage	39	0.788	0.961
Ligament	39	0.635	0.821
Bone	39	0.741	0.682
Interface	39	0.749	0.749

For all the correlations, *p* < 0.001

R1: expert radiologist with 20 years of experience; R2,3: two radiologists with 4 years of experience

*HR-DLR* High-resolution and deep learning reconstructed sequences, *SR* Standard-resolution routine sequences

### Comparison of qualitative scores

**Fig. 2:**
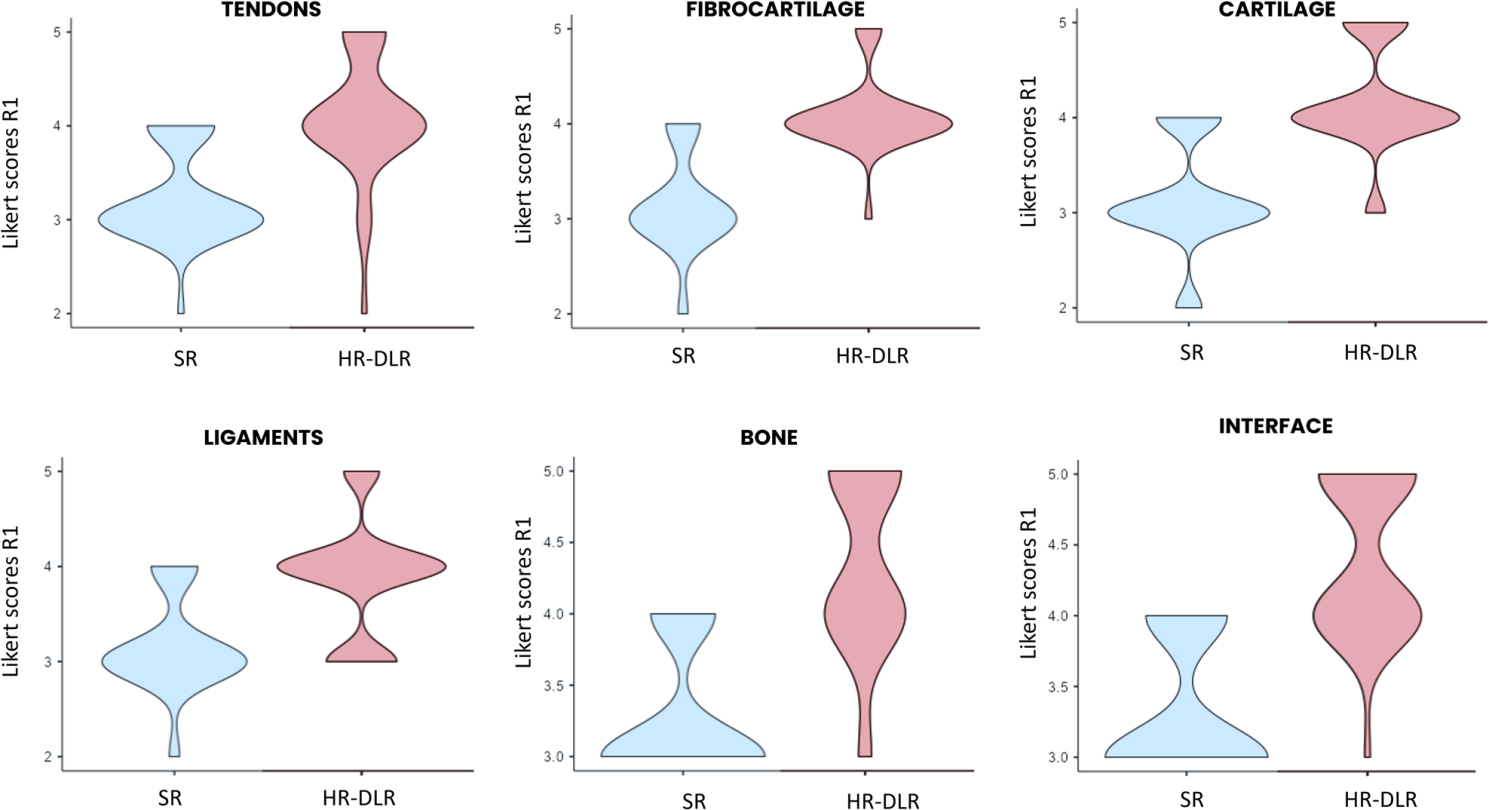
Violin plots of the qualitative assessment (Likert score), regarding the visibility of the different structures (tendons, fibrocartilage, cartilage, ligaments, bone, interface) using the SR and HR-DLR sequences. Values obtained from the 39 subjects, evaluations by the R1. The DLR fixed effect was always significant, with greater HR-DLR scores compared to those from SR images (*p* < 0.001), for all the structures. HR-DLR, High-resolution sequence reconstructed using deep learning; R1, Expert radiologist with 20 years of experience; SR, Standard-resolution routine sequence

Globally, the LMM showed that for each structure, the Likert qualitative diagnostic scores significantly improved in HR-DLR compared to SR sequences. For all the tests, DLR fixed effect was highly significant: (*p* < 0.001), as shown with violin plots in Fig. 2 (R1 evaluations) and Fig. S1 (R2,3 evaluations).

Most interactions between DLR presence and either sequence contrast or joint were not significant, as detailed below, showing a consistent improvement of HR-DLR over SR across sequences and joints (Figs. S2‒S7). The results of each interaction and the *post hoc* pairwise comparisons are reported in the next two paragraphs, separately for the DLR*sequence and DLR*joint interactions. For the few significant interactions, the direction of the effect was always either stable or in favor of HR-DLR sequences.

### Comparison of qualitative scores: interaction between DLR presence and sequence contrast

The interaction between DLR presence and sequence contrast was not significant for the tendon (R1: *p* = 0.142; R2,3: *p* = 0.084; Fig. S2c, d), fibrocartilage (R1: *p* = 0.585; R2,3: *p* = 0.148; Fig. S3c, d), cartilage (R1: *p* = 0.238; R2,3: *p* = 0.644; Fig. S4c, d), ligament R1 (*p* = 0.842; Fig. S5c), bone (R1: *p* = 0.575; R2,3: *p* = 0.223; Fig. S6c, d). Significant interactions were found for the interface (R1: *p* = 0.036; R2,3: *p* = 0.016; Fig. S7c, d), and the ligaments for R2,3 (*p* = 0.001; Fig. S5d). Regarding the interface, the *post hoc* comparisons of SR *versus* HR-DLR sequences, while holding sequence constant, showed that the scores were significantly improved in T1-FSE and PD-FS (*p* < 0.001) sequences, but the comparison did not reach significance in the 3D-T2 sequence (*p* = 0.272 for R1 and *p* = 0.395 for R2,3; Fig. S7c, d). As regards the ligaments for R2,3, they were always significantly better visible in HR-DLR compared to SR sequences for all the contrasts (*p* < 0.001; Fig. S5d).

### Comparison of qualitative scores: interaction between DLR presence and joints

Similarly, only a few interactions between DLR presence and joint were significant: for the ligament (*p* = 0.006 for R1 and *p* < 0.001 for R2,3; Fig. S5a, b) and bone (*p* = 0.011, R1 only; Fig. S6a) scores. The *post hoc* pairwise comparisons of HR-DLR *versus* SR, while holding the joint constant, showed the reasons for the significant interactions, as follows. The ligament scores (Fig. S5a, b) were significantly improved for ankle and knee (*p* < 0.001) for R1 and R2,3, for the hip (*p* = 0.002) according to R2,3 only, but not significantly improved in the shoulder (*p* = 1.0 according to R1 and R2,3) and hip (*p* = 0.213 according to R1). Similarly, the bone R1 scores (Fig. S6a) were significantly improved in the ankle and knee (*p* < 0.001) and hip (*p* = 0.02), but not significantly improved in the shoulder (*p* = 1.000). The interaction between DLR and sequence was not significant for the tendons (R1: *p* = 0.408; R2,3: *p* = 0.641; Fig. S2a, b), fibrocartilage (R1: *p* = 0.906; R2,3: *p* = 0.532; Fig. S3a,b), cartilage (R1: *p* = 0.353; R2,3: *p* = 0.306; Fig. S4a, b), interface (R1: *p* = 0.140; R2,3: *p* = 0.167; Fig. S7a, b) and bone scores given by R2,3 (*p* = 0.272; Fig. S6b).

### SNR and CNR

**Fig. 3:**
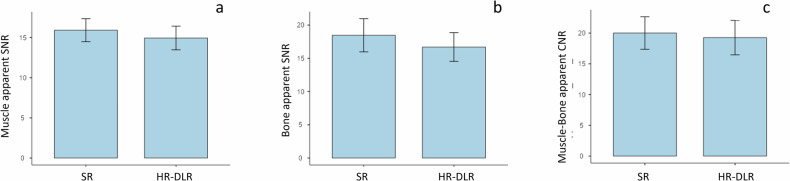
Apparent SNR of the muscle (**a**), apparent SNR of the bone (**b**) and apparent CNR of the muscle *versus* bone (**c**). No significant differences were found between SR and HR-DLR sequences. CNR, Contrast-to-noise ratio; HR-DLR, High-resolution deep learning reconstruction; SNR, Signal-to-noise ratio; SR, Standard resolution

Muscle apparent SNR (*p* = 0.672), bone apparent SNR (*p* = 0.119), muscle-bone apparent CNR (*p* = 0.711) were not significantly different between SR and HR-DLR sequences (Fig. 3). No significant interaction was found between DLR presence and joint for muscle apparent SNR (*p* = 0.309), bone apparent SNR (*p* = 0.077), and apparent CNR (*p* = 0.064) or sequence (*p* = 0.750, 0.638, and 0.331, respectively).

### Diagnostic quality improvement with HR-DLR

**Fig. 4:**
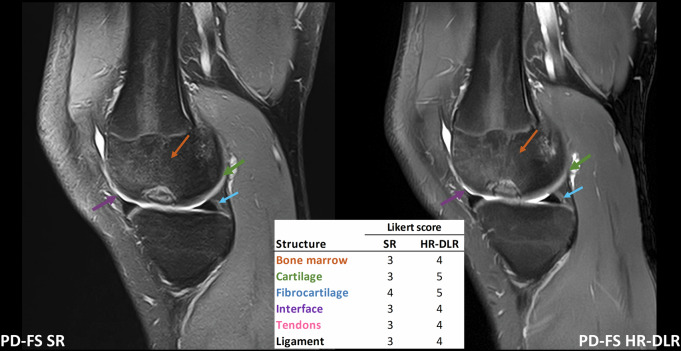
Case 1: Example of an osteochondral lesion of the medial femoral condyle, visualized on sagittal PD-FS images. A comparison is shown between SR sequences (left) and HR-DLR sequences (right). The margins of the lesion are better delineated in the HR-DLR image. Notably, the Likert scale scores were higher for all structures in the HR-DLR sequence compared to the SR (R1 assigned the following scores, reported as SR/HR-DLR. Bone: 3/4; cartilage: 3/5; fibrocartilage: 4/5; interface: 3/4; tendons: 3/4; ligaments: 3/4). HR-DLR, High-resolution deep learning reconstruction; PD-FS, Proton density-weighted sequence with fat saturation; R1, Expert radiologist with 20 years of experience; SR, Standard-resolution routine sequence

**Fig. 5:**
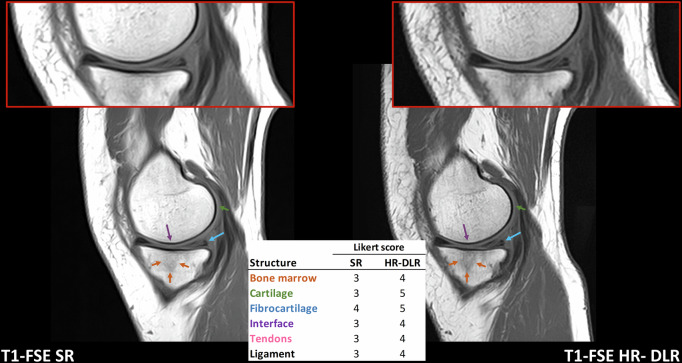
Case 2: Delaminative lesion of the posterior horn of the medial meniscus and subchondral bone edema, better defined in sagittal T1-FSE HR-DLR images (right) than in SR ones (left). The HR-DLR sequence allows to observe the bone edema of the tibial plateau with a better contrast compared to the SR sequence, although fluid-sensitive sequences are electively needed for its proper evaluation. The Likert scores of all the structures are higher in HR-DLR compared to SR (R1 assigned the following scores, reported as SR/HR-DLR. Bone: 3/4; cartilage: 3/5; fibrocartilage: 4/5; interface: 3/4; tendons: 3/4; ligaments: 3/4). HR-DLR, High-resolution deep learning reconstruction; T1-FSE, T1-weighted fast spin-echo; R1, Expert radiologist with 20 years of experience; SR, Standard-resolution routine sequence

**Fig. 6:**
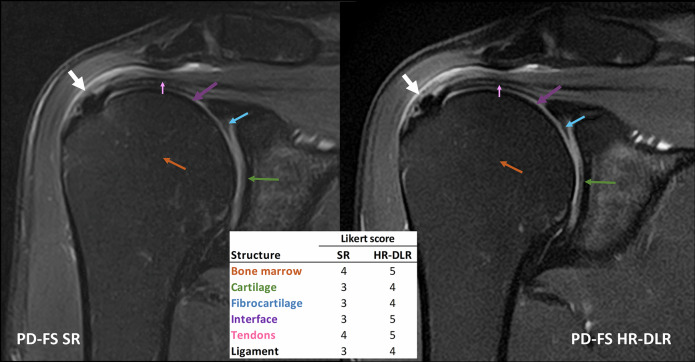
Case 3: Intratendinous calcification of the supraspinatus tendon (pointed with white arrows), visible on coronal PD-FS SR *versus* PD-FS HR-DLR. The DLR sequence allows for a clearer delineation of the calcific formation’s shape and margins. The Likert scores assigned by R1 were the following, reported as SR/HR-DLR. Bone: 4/5; cartilage: 3/4; fibrocartilage: 3/4; interface: 3/5; tendons: 4/5; ligaments: 3/4). HR-DLR, High-resolution deep learning reconstruction; PD-FS, Proton density-weighted sequence with fat saturation; R1, Expert radiologist with 20 years of experience; SR, Standard-resolution routine sequence

**Fig. 7:**
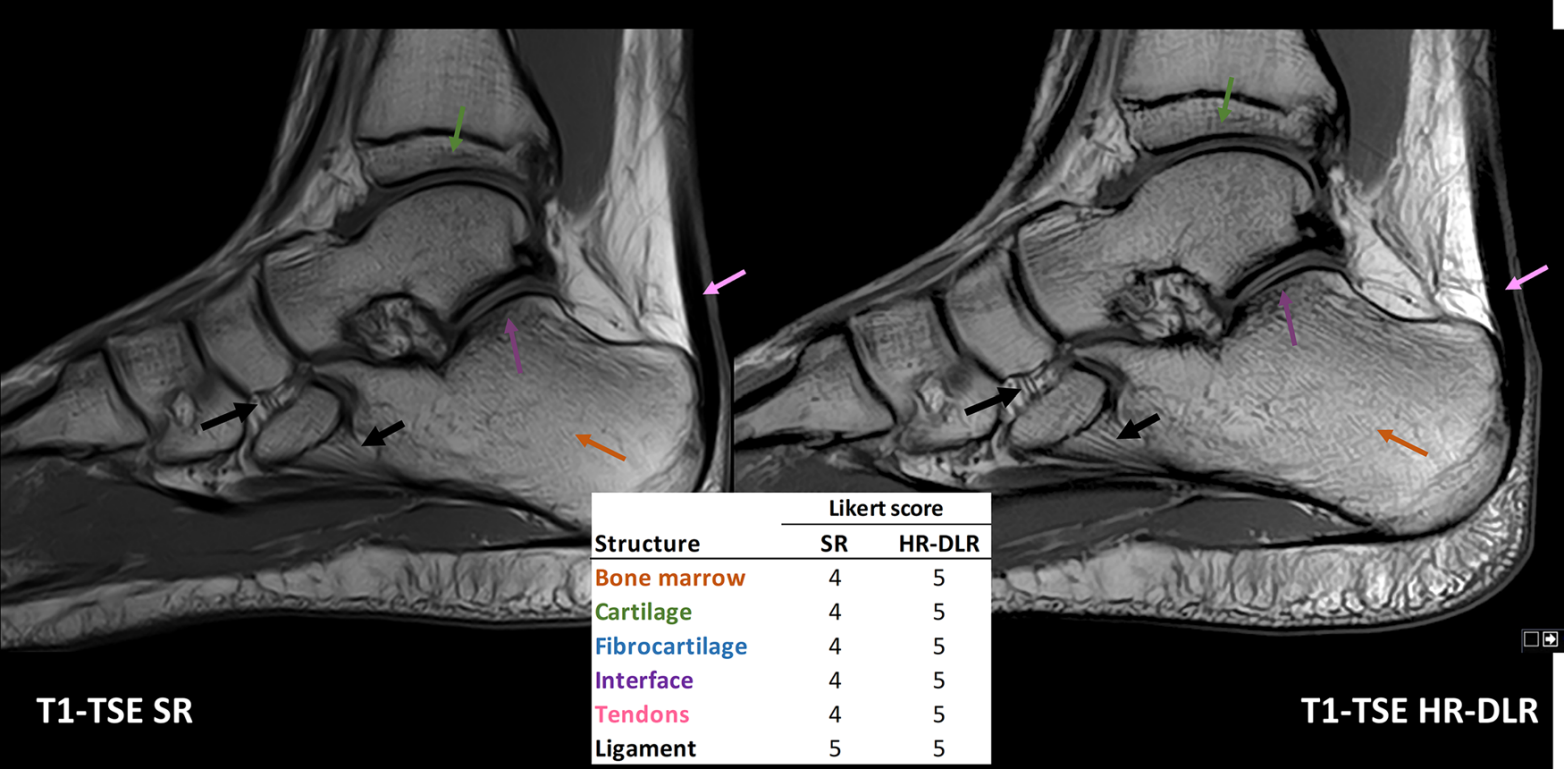
Case 4: right ankle. The scapho-cuboid (left black arrow) and calcaneal-cuboid (right black arrow) ligaments can be better appreciated in the sagittal T1-FSE HR-DLR images compared to the SR ones. All the Likert scores were higher in the HR-DLR sequence compared to the SR one (R1 assigned the following scores, reported as SR/HR-DLR. Bone: 4/5; cartilage: 4/5; fibrocartilage: 4/5; interface: 4/5; tendons: 4/5; ligaments: 5/5). HR-DLR, High-resolution deep learning reconstruction; R1, Expert radiologist with 20 years of experience; SR, Standard-resolution routine sequence; T1-FSE, T1-weighted fast spin-echo

**Fig. 8:**
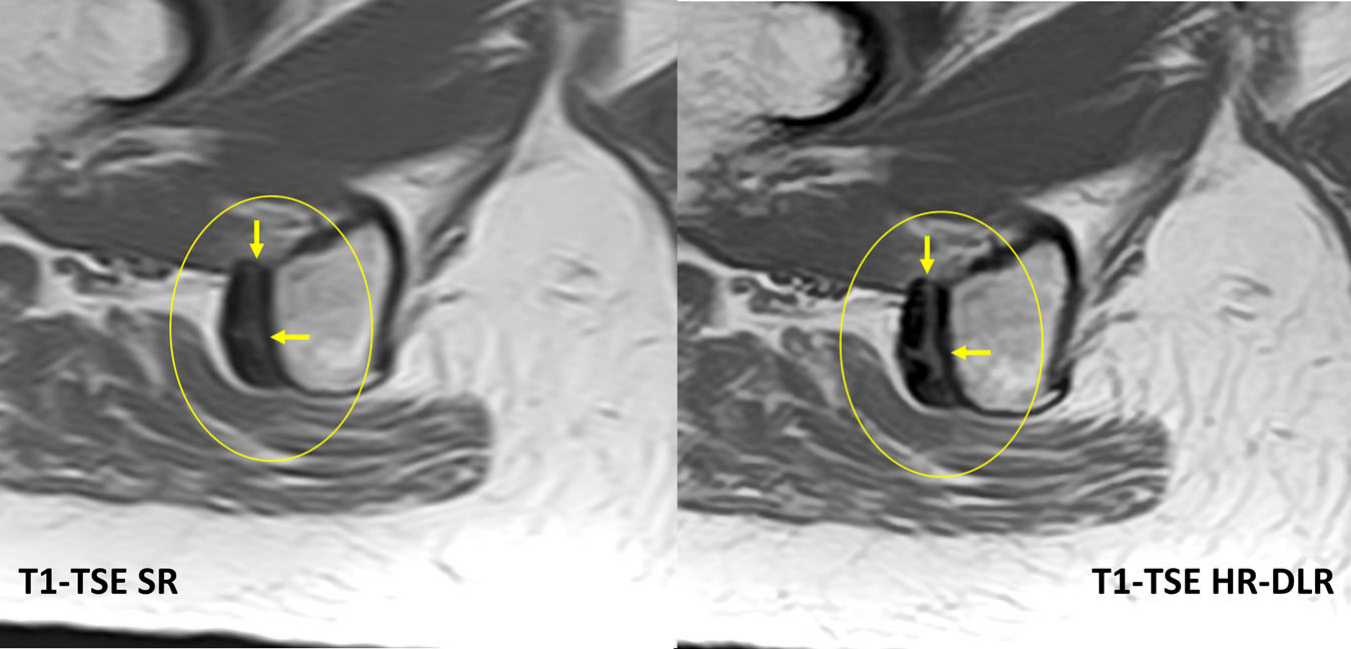
Case 5: Axial T1 SR *versus* T1-DLR. Detailed axial view of the right ischial tuberosity (magnified crop showing tendons, bone marrow, and the interface). Yellow arrows indicate the Hamstring tendon at its origin, which is more clearly visualized in the HR-DLR image. Likert scores of 4 were assigned to the bone, cartilage, interface, tendons, and ligaments in the SR sequence, whereas scores of 5 were assigned to the HR-DLR images. DLR, Deep learning reconstruction; SR, Standard routine sequence as reference; T1-FSE, T1-weighted fast spin-echo

Examples of DRL improved diagnostic confidence or anatomical detail visibility are shown for five cases. For three of them (Figs. 4–6), the pathological findings are better evaluable on HR-DLR images. For the other two cases, some normal anatomical details can be better appreciated on HR-DLR images (Figs. 7, 8).

## Discussion

In this study, DLR improved the radiological visibility of various MSK structures, enhancing the resolution of MRI sequences commonly used for MSK examinations (FSE T1, PD-FS, and FASE 3D T2 sequences). The extent of pixel size reduction, combined with DLR, allowed preservation of SNR and CNR. The 1.5-T scanner was equipped with AiCE, a deep learning neural network designed to reduce image noise [26].

A notable finding was the high inter-rater agreement, despite different levels of reader experience. Importantly, inter-rater concordance was consistently equal or higher for HR-DLR images compared to SR routine sequences, supporting the robustness and reproducibility of the qualitative improvements.

Our results confirm previous studies showing that scan time can be reduced while maintaining or improving image quality [14–20]. The acquisition time reduction of about 4% for the shoulder, and notably of about 22% in the hip and 24% in the ankle (Table 1) has various advantages, improving patient comfort and reducing motion artifacts. In our cohort, one patient showed motion artifacts in the SR PD-FS sequence, while none were observed in HR-DLR sequences. Shorter scan times may also contribute to workflow efficiency and reduced environmental impact. Beyond scan time reduction, DLR was leveraged to increase spatial resolution, similarly to what was reported by Do et al [10].

As shown in Table 1, the shoulder mainly benefited from DLR through a marked resolution increase (41%), with minimal acquisition time reduction (3.8%). Conversely, the hip and ankle showed simultaneous gains in both resolution and acquisition time, exceeding 20%. The knee demonstrated a 12% resolution increase with a negligible scan-time change (+1.9%, 11 s). As detailed in Table S1, this resulted from a 23-s increase in the T1-weighted sequence balanced by a 12-s reduction in PD acquisition time, while FASE 3D-T2 remained unchanged.

Quantitative SNR and CNR were computed as described by Do et al [11]. The absence of significant differences between HR-DLR and SR images indicates that the selected acquisition parameters allowed compensation for potential SNR loss due to a smaller voxel size. This likely reflects the more moderate pixel size reduction compared to the 48% decrease reported in the previously cited study [10].

Consistent with previous studies [14–20, 22, 31–33], DLR improved image quality and diagnostic confidence. The novelties of our study lie in its focus on a 1.5-T scanner and in the evaluation of various sequence contrasts and anatomical joints, contrary to previous published works focusing on a single contrast [19, 24] or joint only [14, 15, 18, 24, 32, 33]. This allowed us to assess the potential influence of sequence contrast and joint type on the various qualitative and quantitative parameters, leading to several observations, as follows.

Different coil configurations were used depending on the anatomical region. Although the present study was not designed to isolate the independent effect of coil channel number, quantitative SNR and CNR measurements did not significantly differ across joints in either SR or DLR images. This suggests that potential coil-related baseline SNR differences were unlikely to drive the observed variability in qualitative scores. Differences in the magnitude of DLR qualitative benefit across joints may instead reflect anatomical complexity and perceptual factors inherent to each region.

For qualitative assessment, most DLR-by-joint and DLR-by-contrast interactions were not significant, indicating consistent improvement across anatomical sites and sequences. Where interactions were significant, *post hoc* analyses showed that HR-DLR images were either equal to or superior to SR; importantly, no MSK structure was rated worse with DLR. Interface visibility improved in HR-DLR images for T1- and PD-weighted sequences (Fig. S7c, d), whereas no significant difference was observed for FASE 3D-T2, possibly due to the limited number of cases (*n* = 7).

For joint-specific analyses (Figs. S2–S7), all evaluated structures were either better or equally visible with HR-DLR. Significant DLR-by-joint interactions for ligaments and bone (R1) indicate that the magnitude of improvement varied across joints, but without deterioration in any comparison.

The used DLR was the same as the previously cited MSK studies [10, 19, 20, 24], *i.e*., AiCE by Canon Medical Systems Corporation. AiCE was recently applied to improve image quality using 1.5-T and 3-T scanners, also in other anatomical areas, such as prostate [34], pelvis [8], brain [26, 35], abdomen [36], and angiography [37].

To our knowledge, this is the first study evaluating multiple joints and comparing routine MSK sequences with HR sequences and at 1.5 T, also including pathological cases. Building upon previous 1.5-T studies [19, 20, 24], we implemented a more moderate resolution increase to preserve SNR.

Although diagnostic confidence was not formally measured, illustrative cases demonstrated improved visualization of pathological structures and anatomical details. Detecting osteochondral lesions, as in Case 1 (Fig. 4), is clinically highly relevant. Accurate assessment of articular cartilage damage on routine MRI has been emphasized in a review [38]: HR-DLR may enhance routine image quality for clearer lesion identification.

HR-DLR visualization of a posterior horn medial meniscus lesion in Case 2 (Fig. 5), one of the most common knee injuries. Early detection is crucial for preventing further joint damage and preserving function [39]. Improving lesion visibility is useful for better discrimination between delaminative lesions from degenerative ones [40], with consequent clinical-surgical impact.

Regarding Case 3, supraspinatus tendon calcification, usually better detectable in radiograms, CT and ultrasound, was also visible in HR-DLR MRI (Fig. 6). Early detection [41], even with MRI, supports timely treatment, such as barbotage, preventing articular and tendinous damages, generating painful crises. A recent paper showed that DL may reduce acquisition time and improve image quality, with similar diagnostic confidence compared with standard MRI in the shoulder [42].

Cases 4 and 5 have no clinically relevant imaging findings, but even in these cases (Figs. 7, 8), the image quality improvement with DLR may support a greater definition of anatomical structures such as tendons and ligaments, especially in trauma cases [1].

The primary limitation of this study is the relatively small sample size. Nonetheless, the main effect of DLR was highly significant across structures. While some interaction analyses showed non-significant differences in subgroups, HR-DLR images were consistently equal or superior to SR images. Additional limitations include the retrospective, single-center design and the absence of detailed clinical indications, limiting assessment of clinical impact. Prospective, multicenter studies would help confirm generalizability. Inclusion of patients with implants or prostheses would further broaden applicability.

In conclusion, we propose a 1.5-T MSK MRI protocol combining higher resolution with overall reduced acquisition time, achieved without compromising SNR or CNR through DLR. As AiCE is integrated into the scanner workflow, it can be directly implemented in routine clinical practice. Our findings support the role of DLR in improving efficiency and structural visualization in 1.5-T MSK MRI.

## Supplementary information

**Additional File: Fig. S1.** Violin plots of the qualitative assessment (Likert score), regarding the visibility of the different structures (tendons, fibrocartilage, cartilage, ligaments, bone, interface) using the SR and HR-DLR sequences. Values obtained from the 39 subjects, evaluations by the R2,3. The DLR fixed effect was always significant, with HR-DLR scores always significantly higher compared to those from SR images (*p* < 0.001) for all the structures. **Fig. S2.** Violin plots of the qualitative assessment (Likert score) regarding the visibility of the tendons, in the 39 subjects evaluated by R1 (**a, c**) and R2,3 (**b, d**) in consensus. The DLR fixed effect was significant in all the cases (*p* < 0.001), regardless joint and sequence contrast, with higher values for the HR-DLR images. The SR *versus* HR-DLR comparisons holding joint constant have been reported in **a**) and **b**); holding sequence contrast constant have been reported in **c**) and **d**). **Fig. S3.** Violin plots of the qualitative assessment (Likert score) regarding the visibility of the fibrocartilage, in the 39 subjects evaluated by R1 (**a**, **c**) and R2,3 (**b**, **d**) in consensus. The DLR fixed effect was significant in all the cases (*p* < 0.001), regardless joint and sequence contrast, with higher values for HR-DLR images. The SR *versus* HR-DLR comparisons holding joint constant have been reported in a) and b); holding sequence contrast constant have been reported in **c**) and **d**). **Fig. S4.** Violin plots of the qualitative assessment (Likert score) regarding the visibility of the cartilage, in the 39 subjects evaluated by R1 (**a**, **c**) and R2,3 (**b**, **d**), in consensus. The DLR fixed effect was significant in all the cases (*p* < 0.001), regardless joint and sequence contrast, with higher values for the HR-DLR images. The SR *versus* HR-DLR comparisons holding joint constant have been reported in **a**) and **b**); holding sequence contrast constant have been reported in **c**) and **d**). **Fig. S5.** Violin plots of the qualitative assessment (Likert score) regarding the visibility of the ligament, in the 39 subjects evaluated by R1 (**a**, **c**) and R2,3 (**b**, **d**) in consensus. The DLR fixed effect was significant in all the cases (*p* < 0.001). TheSR *versus* HR-DLR comparisons holding joint constant have been reported in a) and b); holding sequence contrast constant have been reported in **c**) and **d**). **Fig. S6.** Violin plots of the qualitative assessment (Likert score) regarding the visibility of the bone, in the 39 subjects evaluated by by R1 (**a**, **c**) and R2,3 (**b**, **d**), in consensus. The DLR fixed effect was significant in all the cases (*p* < 0.001). The SR *versus* HR-DLR comparisons holding joint constant have been reported in **a**) and **b**); holding sequence contrast constant have been reported in **c**) and **d**). **Fig. S7.** Violin plots of the qualitative assessment (Likert score) regarding the visibility of the interface, in the 39 subjects evaluated by by R1 (**a**, **c**) and R2,3 (**b**, **d**) in consensus. The DLR fixed effect was significant in all the cases (*p* < 0.001). The SR *versus* HR-DLR comparisons holding joint constant have been reported in **a**) and **b**); holding sequence contrast constant have been reported in **c**) and **d**). **Table S1.** Sequence parameters for the standard resolution (SR) sequences (#) and the high-resolution deep-learning reconstruction (HR-DLR) sequences (§).

## Data Availability

Data may be made available upon reasonable request.

## Abbreviations

AiCE: Advanced intelligent Clear IQ Engine
CNR: Contrast-to-noise ratio
DLR: Deep learning reconstruction
FASE: Fast advanced spin-echo
FS: Fat saturation
FSE: Fast spin-echo
HR: High-resolution
LMM: Linear mixed models
MRI: Magnetic resonance imaging
MSK: Musculoskeletal
PD: Proton density
R1: Reader 1
R2,3: Readers 2 and 3 (in consensus)
ROI: Region of interest
SNR: Signal-to-noise ratio
SR: Standard resolution
