# Supplementary material for: Enhancing resolution and image quality in musculoskeletal MRI using deep learning reconstruction
Source: Eur Radiol Exp. 2026 May 28;10:78. doi: 10.1186/s41747-026-00743-w (PMC13219709; doi:10.1186/s41747-026-00743-w)
Supplement: Supplementary file 1 — Additional File: Fig. S1. Violin plots of the qualitative assessment (Likert score), regarding the visibility of the different structures (tendons, fibrocartilage, cartilage, ligaments, bone, interface) using the SR and HR-DLR sequences. Values obtained from the 39 subjects, evaluations by the R2,3. The DLR fixed effect was always significant, with HR-DLR scores always significantly higher compared to those from SR images (p < 0.001) for all the structures. Fig. S2. Violin plots of the qualitative assessment (Likert score) regarding the visibility of the tendons, in the 39 subjects evaluated by R1 (a, c) and R2,3 (b, d) in consensus. The DLR fixed effect was significant in all the cases (p < 0.001), regardless joint and sequence contrast, with higher values for the HR-DLR images. The SR versus HR-DLR comparisons holding joint constant have been reported in a) and b); holding sequence contrast constant have been reported in c) and d). Fig. S3. Violin plots of the qualitative assessment (Likert score) regarding the visibility of the fibrocartilage, in the 39 subjects evaluated by R1 (a, c) and R2,3 (b, d) in consensus. The DLR fixed effect was significant in all the cases (p < 0.001), regardless joint and sequence contrast, with higher values for HR-DLR images. The SR versus HR-DLR comparisons holding joint constant have been reported in a) and b); holding sequence contrast constant have been reported in c) and d). Fig. S4. Violin plots of the qualitative assessment (Likert score) regarding the visibility of the cartilage, in the 39 subjects evaluated by R1 (a, c) and R2,3 (b, d), in consensus. The DLR fixed effect was significant in all the cases (p < 0.001), regardless joint and sequence contrast, with higher values for the HR-DLR images. The SR versus HR-DLR comparisons holding joint constant have been reported in a) and b); holding sequence contrast constant have been reported in c) and d). Fig. S5. Violin plots of the qualitative assessment (Likert score) regardi [file 41747_2026_743_MOESM1_ESM.pdf]

Enhancing resolution and image quality in musculoskeletal MRI using deep learning reconstruction

ELECTRONIC SUPPLEMENTARY MATERIAL

Deep learning reconstruction (DLR) using Advanced Intelligent Clear IQ Engine (AiCE)

Raw data acquired from the 1.5-T scanner were reconstructed using the vendor-provided deep learning reconstruction (DLR) Advanced Intelligent Clear IQ Engine (AiCE).

The algorithm implements a Convolutional Neural Network that was trained on paired datasets of noisy input images and high signal-to-noise ratio (SNR) ground-truth reference images [1–4]. During training, the Convolutional Neural Network learned the parameters necessary to suppress Gaussian white noise and restore lost fine structures, thereby generating sharp, clear images while preserving the anatomical information.

The reconstruction pipeline is implemented after conversion of k-space data to real-space data (the image domain) using an inverse fast Fourier transform procedure. The Convolutional Neural Network - based denoising step is then applied to this image domain.

Specifically, the DLR method involves the following steps:

1. Feature extraction: The input image data undergoes convolution using a 7×7 Discrete Cosine Transform kernel. This divides the image data into a low-frequency component (which helps maintain image contrast) and other high-frequency components. The low-frequency component follows a separate collateral path.

2. Denoising: The high-frequency components are processed through subsequent feature conversion layers (22 layers). Within these layers, a soft-shrinkage activation function is applied for adaptive denoising suitable for various noise levels.

3. Final image Generation: The final denoised image is obtained by performing deconvolution using a 7×7 inverse Discrete Cosine Transform kernel on the noise-reduced data, combined with the low-pass filtered low-frequency component.

4. Based on a MAP scan at the start of the examination the system determines expected noise levels and uses this for the level of denoising that is applied. The user can overrule this by applying an AiCE adjustment factor (between 0.7 – 3.0)

5. The final image output by the denoising module is a blend between the original, native image and denoised image with ratios as DLR levels presets (d01 – d05), where a d01 blend shows mostly the native image with a limited amount of the denoised image and d05 shows mostly the denoise image and a limited amount of the native image. In this way the look and feel of the images is more natural and can be adjusted to the preference of the radiologist.

The DLR algorithm is a fully integrated reconstruction method, operates automatically, and is CE/FDA certified for clinical use.

The DLR level and adjustment factor used in the study are specified in the Table S1, have been chosen based on previous experiences on similar exams, and on the feedback provided by the radiologists of the clinic during the initial adoption of the sequences in clinical practice.

Supplementary References

1. Tajima T, Akai H, Yasaka K et al (2023) Usefulness of deep learning-based noise reduction for 1.5 T MRI brain images. Clin Radiol 78:e13–e21. https://doi.org/10.1016/j.crad.2022.08.127

2. Ueda T, Ohno Y, Yamamoto K et al (2021) Compressed sensing and deep learning reconstruction for women's pelvic MRI denoising: Utility for improving image quality and examination time in routine clinical practice. Eur J Radiol 134:109430. https://doi.org/10.1016/j.ejrad.2020.109430

3. Ueda T, Ohno Y, Yamamoto K et al (2022) Deep Learning Reconstruction of Diffusion-weighted MRI Improves Image Quality for Prostatic Imaging. Radiology 303:373–381. https://doi.org/10.1148/radiol.204097

4. Akai H, Yasaka K, Sugawara H et al (2022) Commercially Available Deep-learning-reconstruction of MR Imaging of the Knee at 1.5T Has Higher Image Quality Than Conventionally-reconstructed Imaging at 3T: A Normal Volunteer Study. Magn Reson Med Sci 22:353–360. https://doi.org/10.2463/mrms.mp.2022-0020

Supplementary Figures

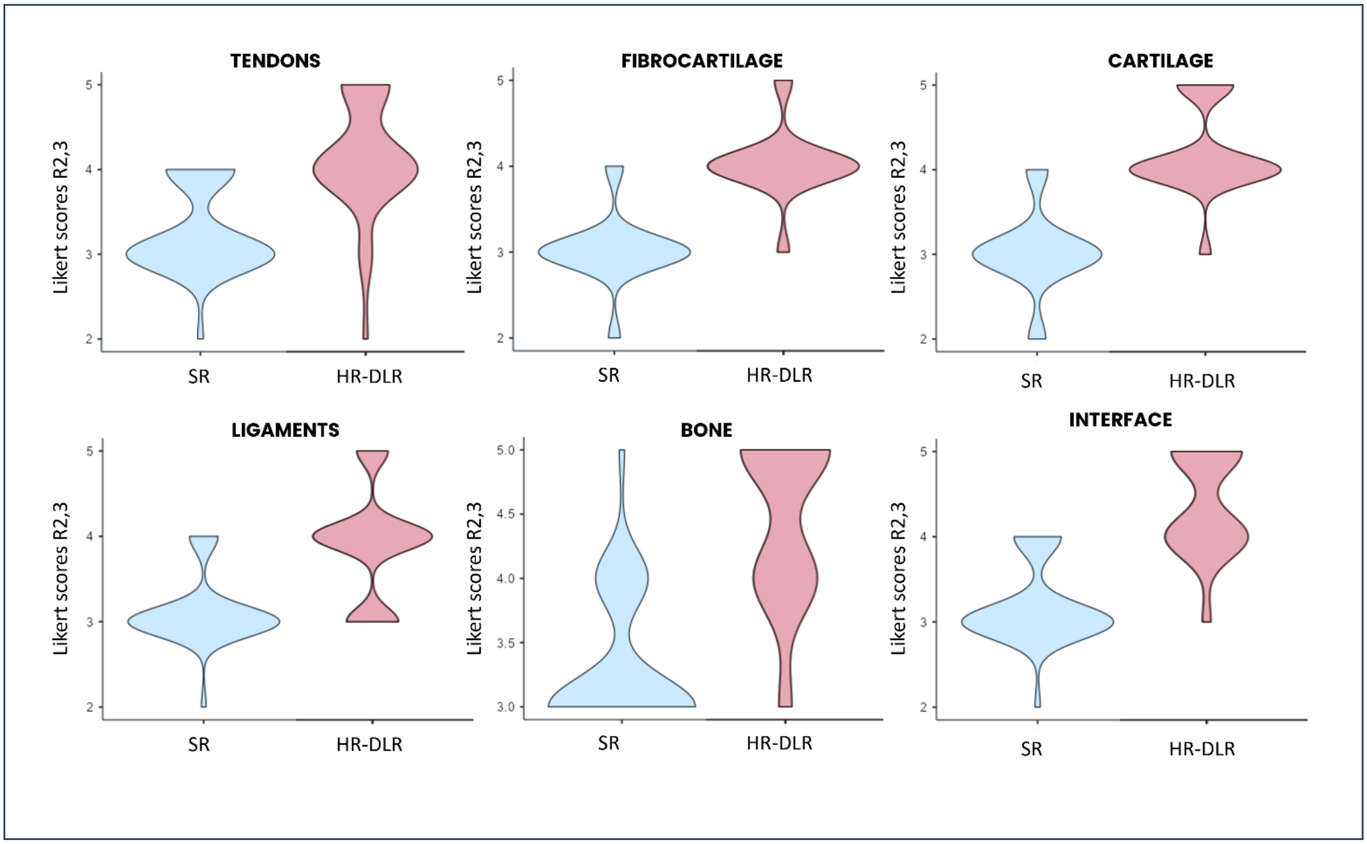

Fig. S1. Violin plots of the qualitative assessment (Likert score), regarding the visibility of the different structures (tendons, fibrocartilage, cartilage, ligaments, bone, interface) using the SR and HR-DLR sequences. Values obtained from the 39 subjects, evaluations by the R2,3. The DLR fixed effect was always significant, with HR-DLR scores always significantly higher compared to those from SR images ($p<0.001$) for all the structures. DLR: Deep Learning Reconstruction; HR: high-resolution; R2,3: two radiologists with 4 years of radiological experience; SR: standard routine sequence as reference.

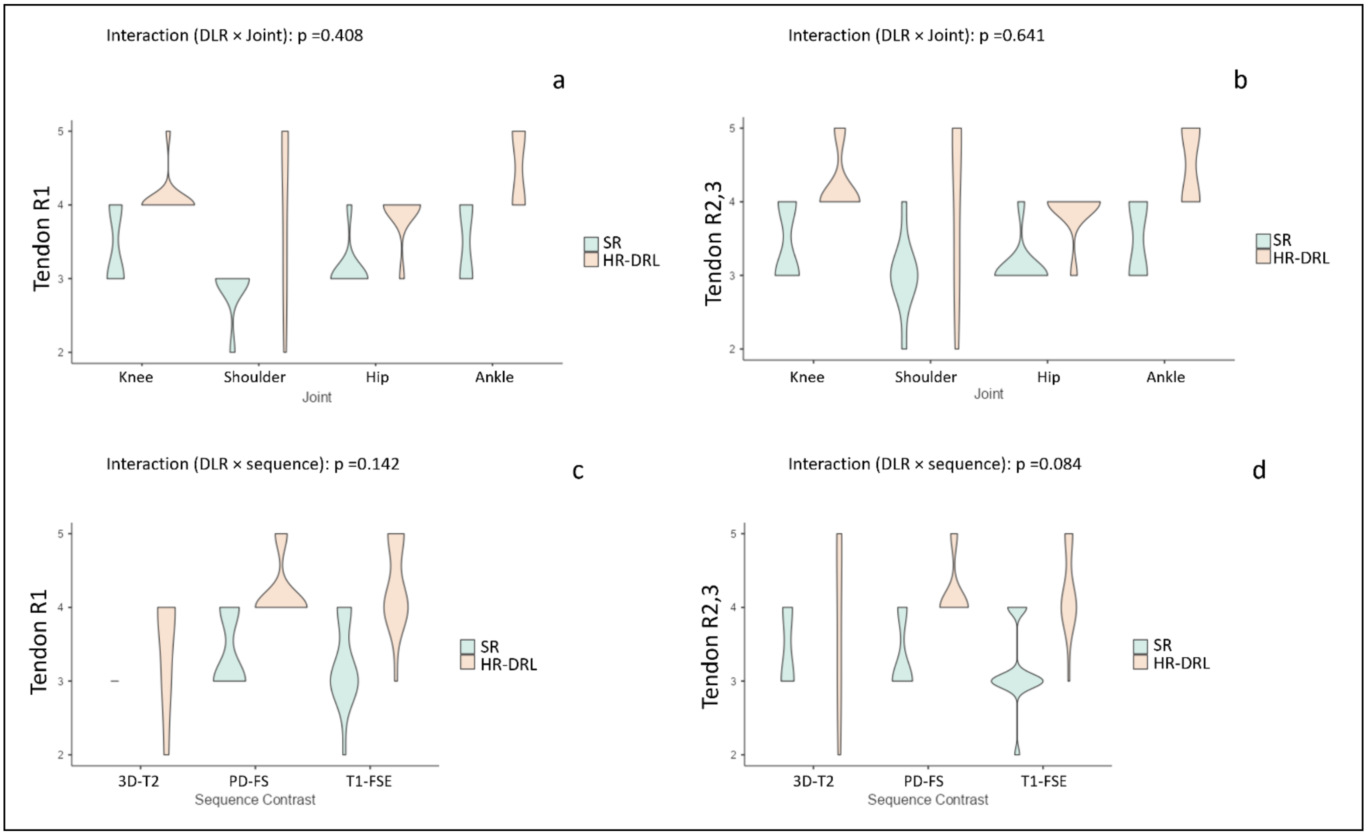

Fig. S2. Violin plots of the qualitative assessment (Likert score) regarding the visibility of the tendons, in the 39 subjects evaluated by R1 (a, c) and R2,3 (b, d) in consensus. The DLR fixed effect was significant in all the cases (p<0.001), regardless joint and sequence contrast, with higher values for the HR-DLR images. The SR *versus* HR-DLR comparisons holding joint constant have been reported in a) and b); holding sequence contrast constant have been reported in c) and d). DLR: Deep Learning Reconstruction; HR: high-resolution; R1; expert radiologist with 20 years of radiological experience; R2,3: two radiologists with 4 years of radiological experience; SR: standard routine sequence as reference.

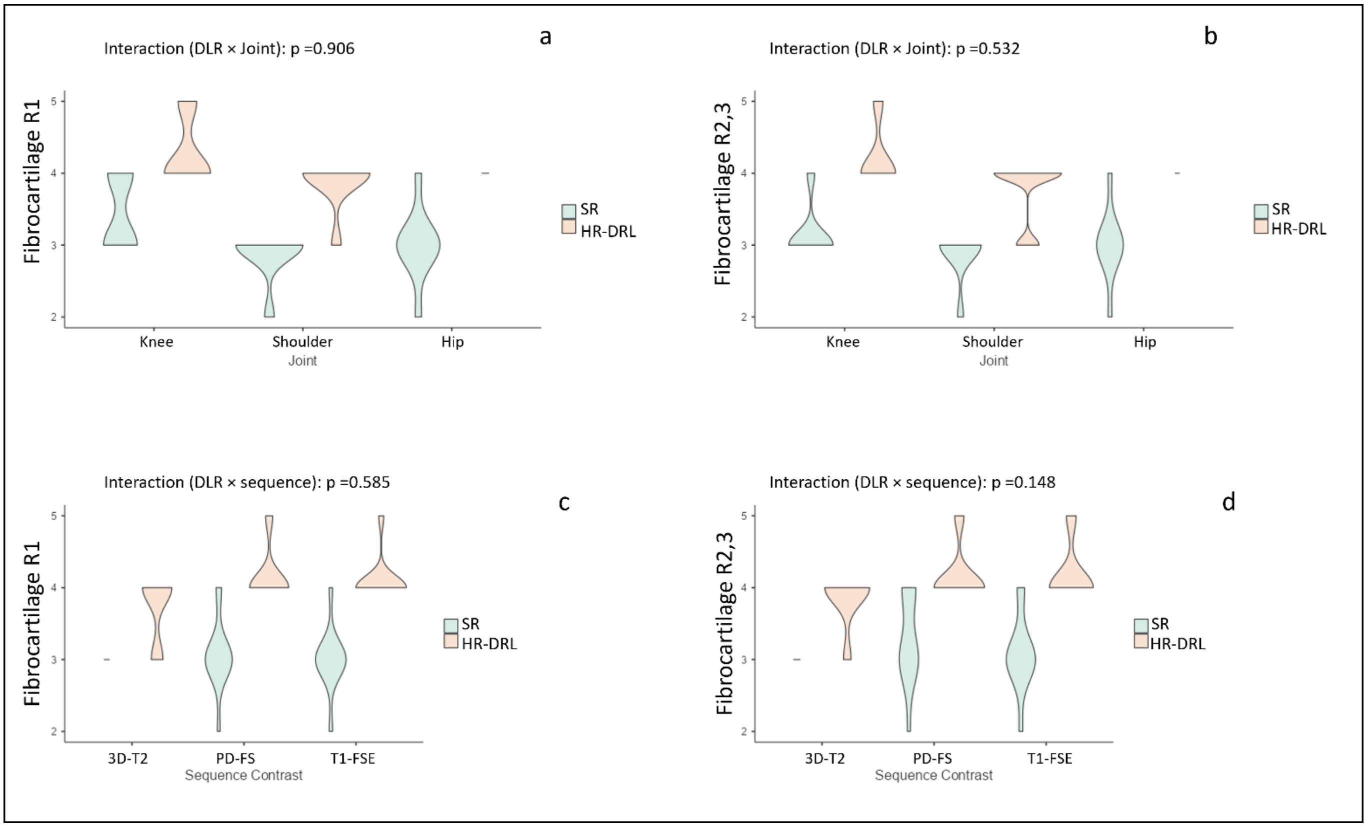

Fig. S3. Violin plots of the qualitative assessment (Likert score) regarding the visibility of the fibrocartilage, in the 39 subjects evaluated by R1 (a, c) and R2,3 (b, d) in consensus. The DLR fixed effect was significant in all the cases (p<0.001), regardless joint and sequence contrast, with higher values for HR-DLR images. The SR *versus* HR-DLR comparisons holding joint constant have been reported in a) and b); holding sequence contrast constant have been reported in c) and d). DLR: Deep Learning Reconstruction; HR: high-resolution; R1; expert radiologist with 20 years of radiological experience; R2,3: two radiologists with 4 years of radiological experience; SR: standard routine sequence as reference.

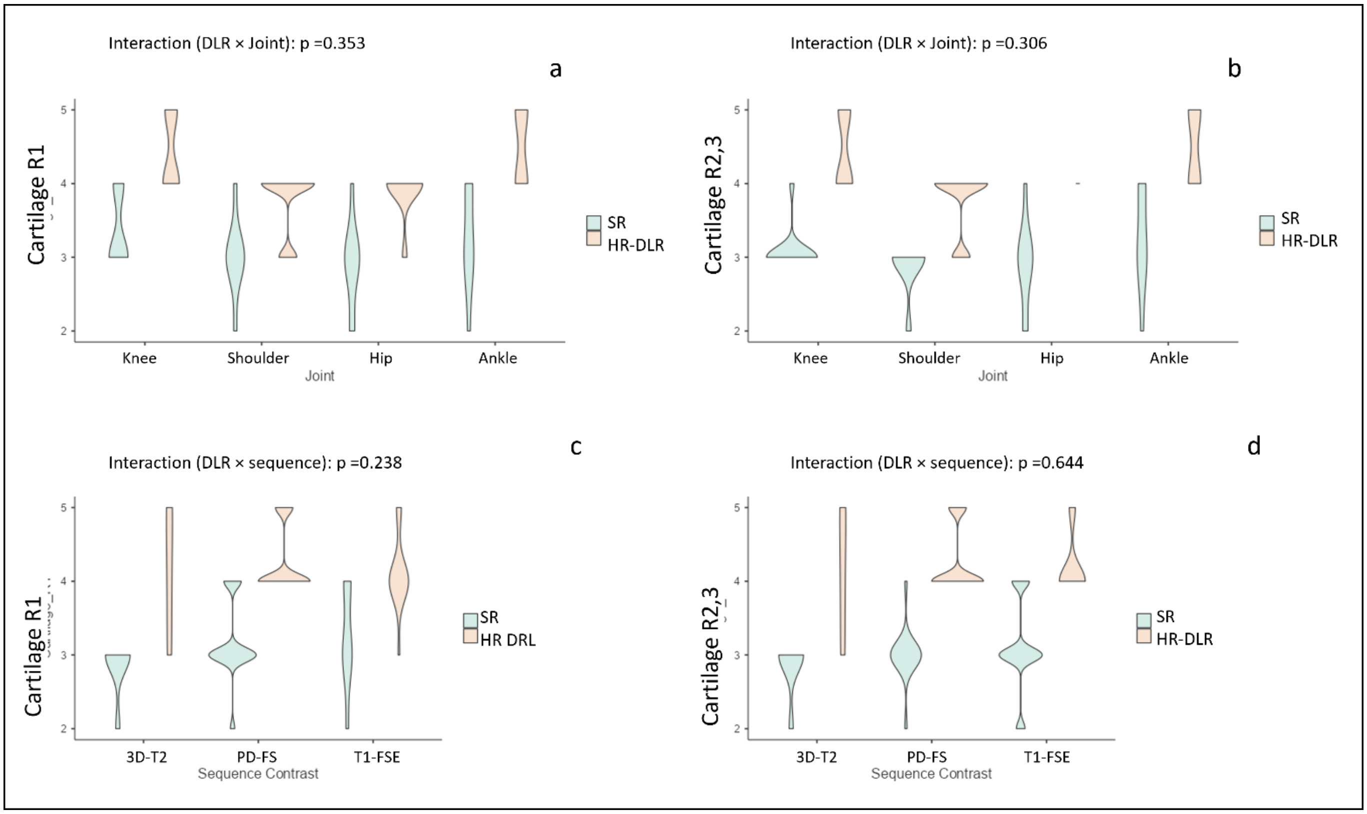

Fig. S4. Violin plots of the qualitative assessment (Likert score) regarding the visibility of the cartilage, in the 39 subjects evaluated by R1 (a, c) and R2,3 (b, d), in consensus. The DLR fixed effect was significant in all the cases ($p<0.001$), regardless joint and sequence contrast, with higher values for the HR-DLR images. The SR *versus* HR-DLR comparisons holding joint constant have been reported in a) and b); holding sequence contrast constant have been reported in c) and d). DLR: Deep Learning Reconstruction; HR: high-resolution; R1; expert radiologist with 20 years of radiological experience; R2,3: two radiologists with 4 years of radiological experience; SR: standard routine sequence as reference.

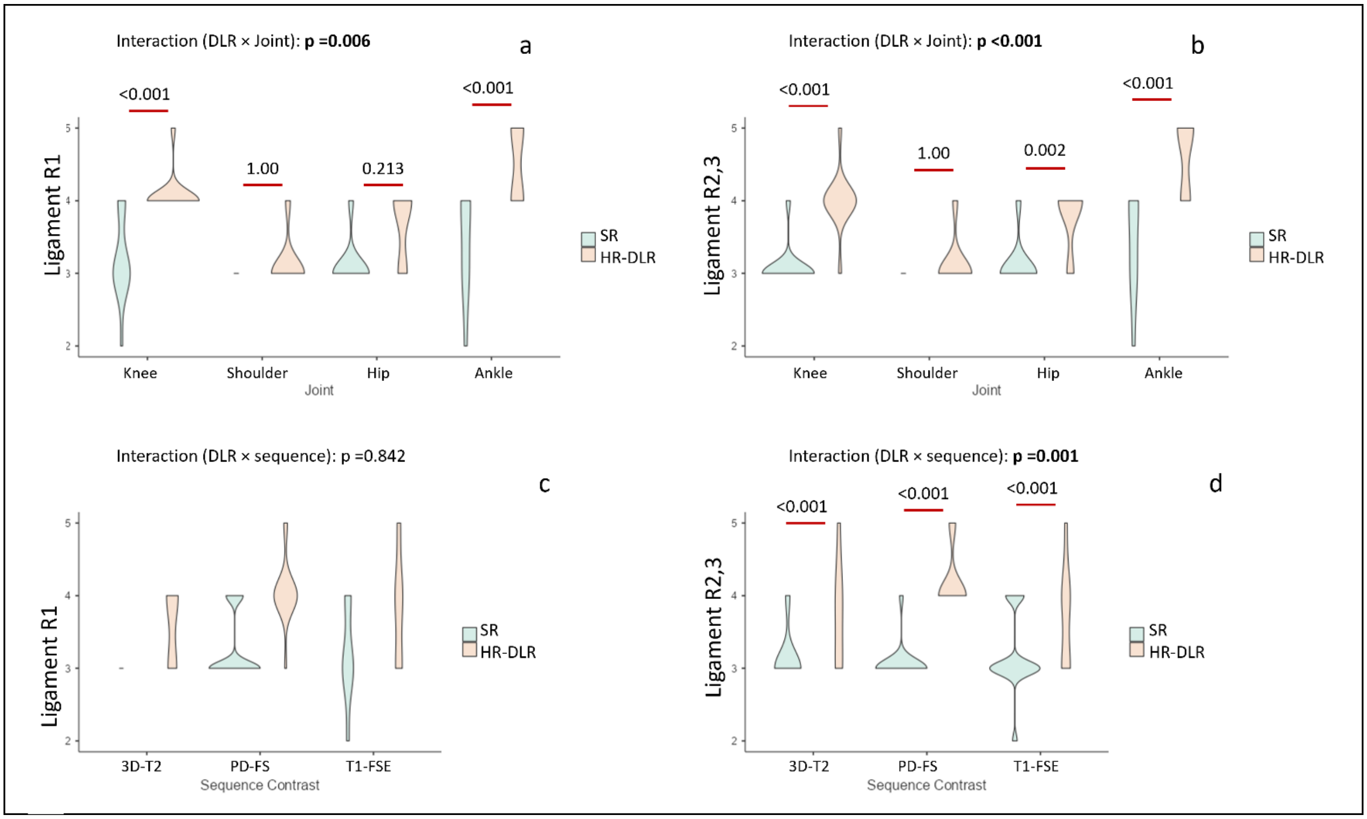

Fig. S5. Violin plots of the qualitative assessment (Likert score) regarding the visibility of the ligament, in the 39 subjects evaluated by R1 (a, c) and R2,3 (b, d) in consensus. The DLR fixed effect was significant in all the cases (p<0.001). TheSR *versus* HR-DLR comparisons holding joint constant have been reported in a) and b); holding sequence contrast constant have been reported in c) and d). DLR: Deep Learning Reconstruction; HR: high-resolution; R1; expert radiologist with 20 years of radiological experience; R2,3: two radiologists with 4 years of radiological experience; SR: standard routine sequence as reference.

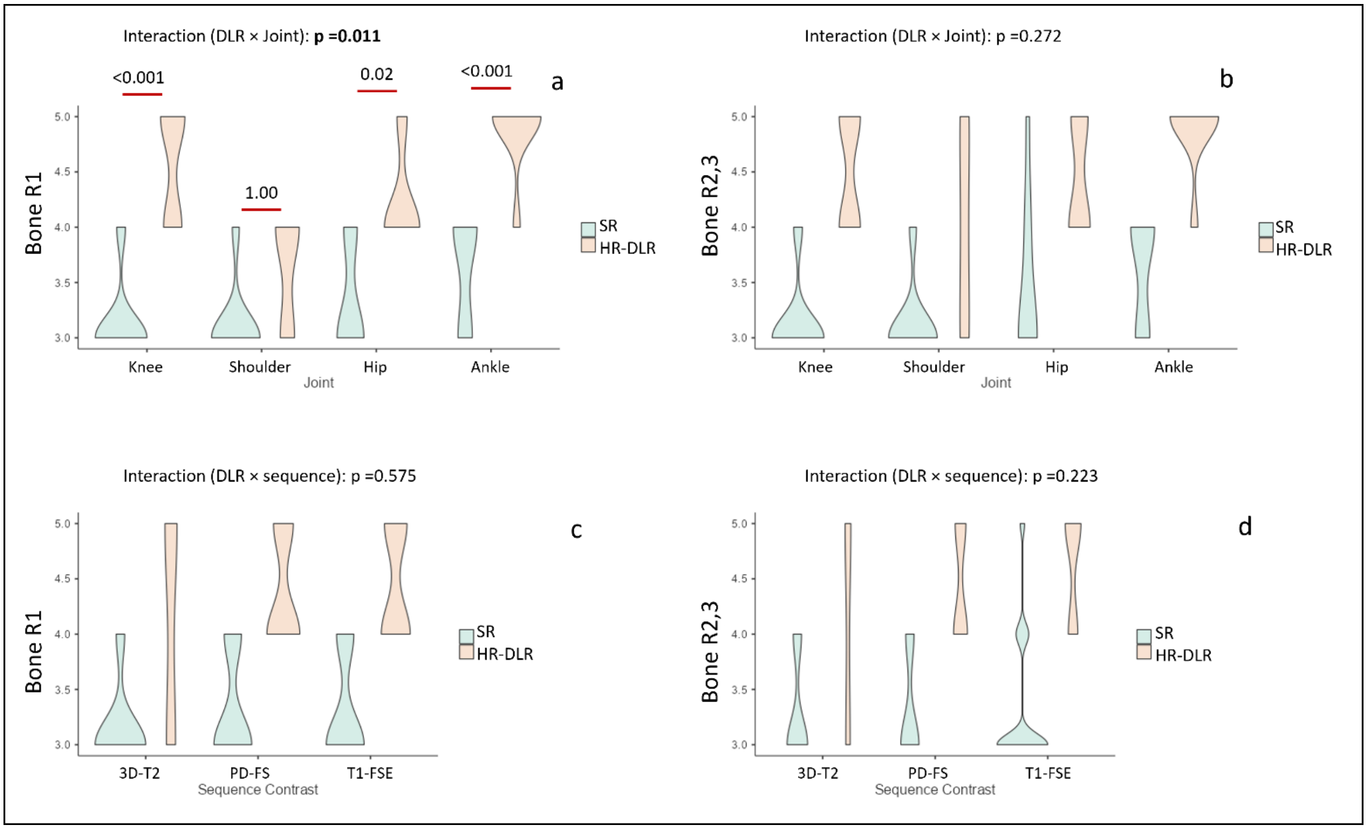

Fig. S6. Violin plots of the qualitative assessment (Likert score) regarding the visibility of the bone, in the 39 subjects evaluated by by R1 (a, c) and R2,3 (b, d), in consensus. The DLR fixed effect was significant in all the cases (p<0.001). The SR *versus* HR-DLR comparisons holding joint constant have been reported in a) and b); holding sequence contrast constant have been reported in c) and d). DLR: Deep Learning Reconstruction; HR: high-resolution; R1; expert radiologist with 20 years of radiological experience; R2,3: two radiologists with 4 years of radiological experience; SR: standard routine sequence as reference.

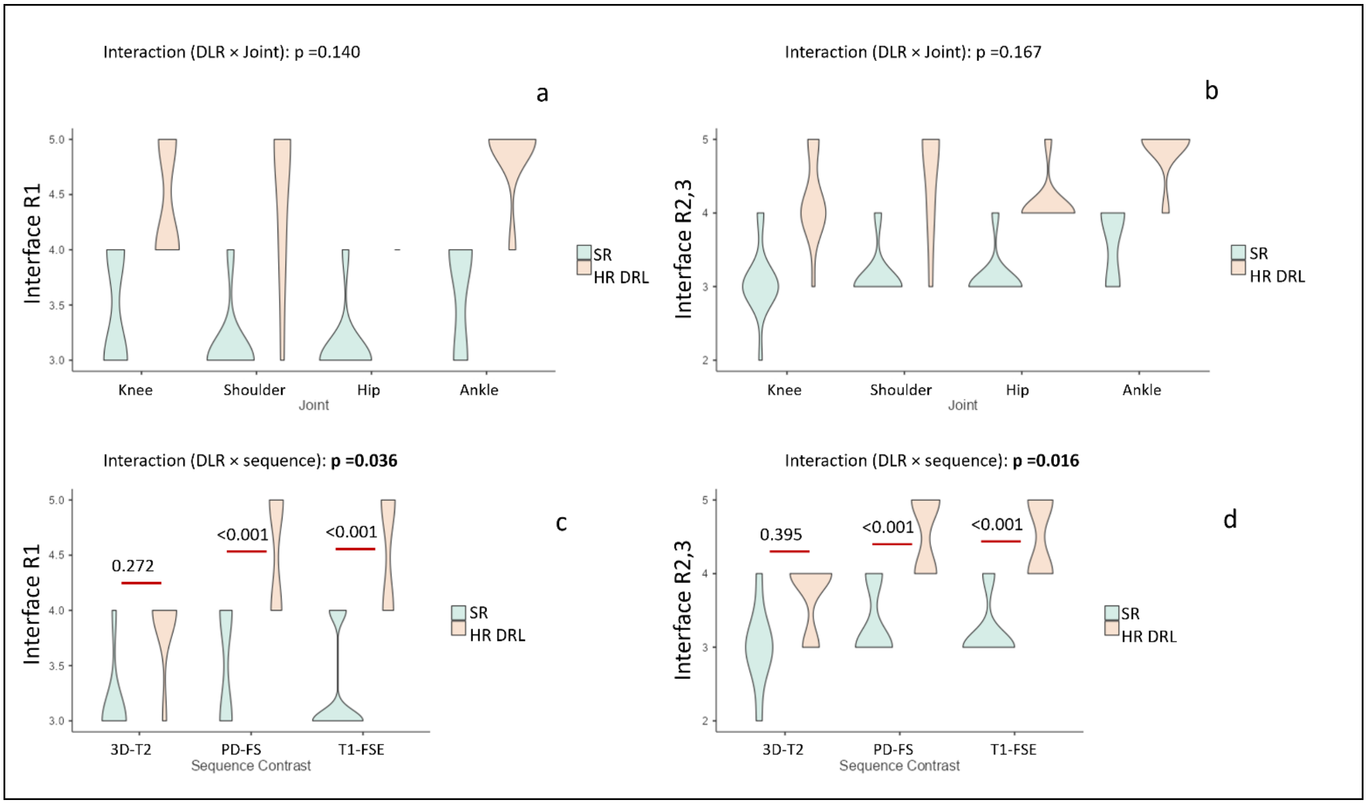

Fig. S7. Violin plots of the qualitative assessment (Likert score) regarding the visibility of the interface, in the 39 subjects evaluated by by R1 (a, c) and R2,3 (b, d) in consensus. The DLR fixed effect was significant in all the cases (p<0.001). The SR *versus* HR-DLR comparisons holding joint constant have been reported in a) and b); holding sequence contrast constant have been reported in c) and d). DLR: Deep Learning Reconstruction; HR: high-resolution; R1; expert radiologist with 20 years of radiological experience; R2,3: two radiologists with 4 years of radiological experience; SR: standard routine sequence as reference.

Table S1. Sequence parameters for the standard resolution (SR) sequences (#) and the high-resolution deep-learning reconstruction (HR-DLR) sequences (§)

Body part	Sequence	Scan plane	Field of view (cm × cm)	Acquisition matrix	Reconstruction matrix	Slice thickness (mm)	TR (ms)	TE (ms)	Flip/flop angle (°)	Echo factor	Type of gain algorithm# or DLR level and adjustment factor§	Acquisition time (s)
Knee	T1-FSE SR	Sagittal	16 × 16	256 × 256	512 × 512	3.0	425	10	90/160	4	GA43	172
	T1-FSE HR-DLR	Sagittal	16 × 16	288 × 288	576 × 576	3.0	656	12	90/180	3	d03 1.5	195
	PD-FS SR	Sagittal	16 × 16	256 × 256	512 × 512	3.0	2450	24	90/160	9	GA54	216
	PD-FS HR-DLR	Sagittal	18 × 18	288 × 288	576 × 576	3.0	3283	36	90/180	13	d03 2.0	204
	FASE 3D-T2 SR	Axial	25 × 25	256 × 256	512 × 512	2.0	2600	297	84/180	2	GA01	198
	FASE 3D-T2 HR-DLR	Axial	17.3 × 17.3	192 × 192	384 × 384	2.0	2000	225	90/180	3	d03 1.5	198
Shoulder	T1-FSE SR	Coronal	19 × 19	224 × 288	448 × 576	3.0	676	10	90/160	2	GA44	132
	T1-FSE HR- DLR	Coronal	19 × 19	352 × 352	704 × 704	3.0	600	12	90/180	3	d03 2.0	158
	PD-FS SR	Sagittal	19 × 19	256X256	512 × 512	3.0	2000	36	90/160	3	GA54	194
	PD-FS HR- DLR	Sagittal	18 × 18	288 × 288	576 × 576	3.0	2200	48	90/180	9	d04 1.5	146
	FASE 3D-T2 SR	Axial	25 × 25	256 × 256	512 × 512	2.0	2600	297	84/180	2	GA01	198
	FASE 3D-T2 HR-DLR	Axial	20.3 × 20.2	224 × 224	448 × 448	1.3	2500	380	90/180	2	d03 1.0	200
Hips	T1-TSE SR	Axial	40 × 28	224 × 320	448 × 640	5.0	755	10	90/160	4	GA54	189
	T1-TSE HR- DLR	Axial	37 × 28	288 × 352	576 × 704	5.0	651	8.5	90/180	4	d03 1.5	180
	PD-FS SR	Coronal	39 × 30	256 × 320	512 × 640	5.0	2800	20	90/160	5	GA54	258
	PD-FS HR- DLR	Coronal	37 × 32	320 × 352	640 × 704	5.0	3000	20	90/180	9	d03 1.5	168
Ankle	T1-TSE SR	Sagittal	17 × 18	288 × 336	512 × 672	3.0	610	10	90/160	2	GA54	159
	T1-TSE HR- DLR	Sagittal	17 × 17	384 × 384	768 × 768	3.0	650	12	90/180	4	d03 2.0	130
	PD-FS SR	Coronal	14 × 18	256 × 352	512 × 704	3.0	2000	30	90/160	6	GA54	206
	PD-FS HR- DLR	Coronal	17 × 17	352 × 352	704 × 704	3.0	2686	36	90/180	9	d03 2.0	148

FASE Fast advanced spin-echo, *FSE* Fast spin-echo, *HR-DLR* High-resolution deep-learning reconstruction, *PD-FS* Proton-density weighted sequence with fat saturation, *TE* Echo time, *TR* Repetition time
